# How registered nurses balance limited resources in order to maintain competence: a grounded theory study

**DOI:** 10.1186/s12912-021-00672-6

**Published:** 2021-09-22

**Authors:** Sharon Rees, Helen Farley, Clint Moloney

**Affiliations:** University of Southern Queensland, Toowoomba, Australia

**Keywords:** Mobile learning, Online learning, Nurse education, Professional development, Continuing education

## Abstract

**Background:**

Nurses have limited time outside of work for continuing professional development. Consequently, strategies need to be explored to enable them to better maintain their competence. This article describes recent research investigating if nursing behaviours in the use of mobile technologies could be leveraged to better facilitate mobile learning. It addresses a gap in the existing literature around how nurses resource their own professional development and learning in the absence of appropriate learning resources in the workplace.

**Methods:**

The research employed a classic grounded theory methodology which was conducted with 27 registered nurses from Public and Private Hospitals in Queensland and external postgraduate nursing students from Victoria, South Australia and the Northern Territory enrolled at the University of Southern Queensland.

**Results:**

The Theory of Economising Learning describes how nurses maintain competence with limited resources. Unfavourable staffing levels and a fast-paced workplace mean that nurses rarely prioritise their professional learning while at work. Instead, it requires the nurse to contribute personal resources including time and money.

Though the research revealed nurses were unconcerned about using mobile technologies, they were concerned about maintaining competence with limited resources. To counter this, nurses economised their learning by balancing personal resources against their motivation to maintain competence. The process of economising learning begins and ends with the development of the nurse’s personal curriculum in response to what they identify as being the most significant knowledge deficits at work that jeopardise their competence. A learning opportunity that addresses the knowledge deficit is sought. Nurses balance the opportunity to address the deficit against the cost of personal resources, to decide if they will engage with the opportunity and update their personal curriculum accordingly.

**Conclusions:**

It is suggested that workplaces need to create reasonable expectations within nurses to address knowledge deficits and provide the resources, including time, to allow them to do so without personal cost. It is also necessary for workplaces to moderate the flow of learning opportunities so as not to overwhelm and demotivate the nurses. Currently, nurses use several strategies to optimise their learning using mobile technologies which could be leveraged in the workplace.

## Background

For nursing to maintain itself as a profession, it is essential to improve the profession, and the standards the profession sets for itself, using evidence-based knowledge [1]. It can be challenging for those within the profession to keep up to date with and utilise the ever-expanding range of innovative care strategies, treatment modalities, and technologies emerging in healthcare [2, 3]. The transition from a newly university-graduated nurse to an effective practitioner can often be difficult. In the university, learning and teaching may be technology-driven and innovative, usually providing good online access to support learning [4–6]. By way of contrast, the nurses’ capacity to continue learning is compromised when they enter the “real world” of nursing. Many practical barriers hinder learning and the supportive environment of the university is no longer evident [7, 8]. Even so, lip-service is given to life-long learning in the profession, especially given the current and pending ageing population with their multiple and complex comorbidities [3, 9, 10]. Continued access to lifelong learning resources is the true problem that justifies a need for research offering insight into sustained, user friendly, and accessible solutions for a nurses continued learning journey [1].

Continuing professional development is essential in fostering a nursing workforce that is up-to-date with best practice, and able to provide quality care to patients through the implementation of new knowledge [11, 12]. As a result, nurses also need the ability to find information quickly when faced with knowledge deficits [13], and the strict requirements for continuing professional development to maintain nursing registration reflect its importance [14, 15]. The ways in which the nursing workforce continues to grow knowledge becomes a vital consideration as nurses adapt to the rapid changes in healthcare. In a climate of increasing workload pressures and staff shortages, traditional classroom teaching models fail to accommodate these emergent needs for knowledge acquisition. Staff shortages and issues with skill mix have meant that nurses are often prevented from engaging with professional learning within normal work hours [16]. With a predicted shortage of nurses into the foreseeable future, this scenario is likely to worsen [10]. Instead, nurses are investing personal time and finances to participate in education outside of their work hours. This impacts on work-life balance, taking them away from the necessary downtime with family and friends [16, 17].

The availability of technologies to enable online learning has rapidly increased over the past decade [5, 18, 19]. The evolution and proliferation of these technologies has happened so quickly that those organisations responsible for delivering education have often struggled to embed and monitor their effectiveness [20]. What is also unclear is the end users’ – in this case nurses’ – preferences for using technologies for learning and teaching. The notion of a knowledgeable lecturer taking centre stage with a room full of deferential students taking notes, asking questions, and having critical debate, is becoming outdated [21]. Instead, they are seeing blended models of learning that combine face-to-face learning with online activities. Ideally, nurses’ learning spaces would supersede the typical classroom of old [22–24], and make extensive use of simulations, artificial intelligence agents, and haptic technologies [25].

Learning mediated by digital technologies such as mobile phones, has the potential to improve nurses’ access to education, with the added advantage of facilitating engagement when and where it is convenient [24, 26, 27]. Mobile learning can occur anywhere and anytime, maximising its potential to happen at a time and place of optimal convenience to the learner [19, 28, 29]. It is rarely the sole delivery mechanism for a unit of learning, as advantages can be maximised by combining it with other forms of interaction [12, 24]. Mobile learning has already been used in discrete projects within nursing and other healthcare professions, mostly with undergraduate learners. The documented successes indicate that it potentially provides benefits for the learner in terms of convenience, interactivity and enhanced communication [28, 30, 31]. The aim of this research was to engage with registered nurses discerning how they were using mobile technologies in their personal and work lives, with a view to leveraging those existing behaviours – if appropriate – to introduce mobile learning for continuing professional development.

## Methods

### Study design

Classic grounded theory, as espoused by Barney Glaser [32–34], was the methodological framework which underpinned this research. Grounded theory was used as it gives the researcher an understanding of the pertinent issues in an area and the behaviours that are used to resolve them. Importantly, it focuses on the interests of the participants [33]. Grounded theory was compatible with the aims of this research, especially given the focus on the issues and behaviours identified by the nurses themselves.

### Participants

Twenty-seven nurses were interviewed over a six-month period, recruited from a wide variety of locations in Australia to ensure as broad a sample as possible. Registered nurses from both private (thirteen) and public health services (six) were recruited from a range of locations including metropolitan (nine), regional (thirteen), rural (three) and remote (two) areas in Queensland (twenty-three), Victoria (one), South Australia (one) and the Northern Territory (one). Twenty-four female and four male participants were included in the research with a large diversity in ages and nursing experience ranging from new graduates to senior nurses with many years’ experience. As the research progressed, it became evident that there was no significant difference in the data elicited from nurses residing in different states. However, there were potential variations in the data depending on the amount of time since graduation, therefore, further participants with varying times since graduation were sought to broaden the data set.

### Ethical considerations

Multi-site ethics approval was gained from Queensland Health and St Vincent’s Hospitals (large private hospitals) within Queensland. Ethics approval was also gained from the University of Southern Queensland which enabled access to students undertaking postgraduate study at this distance-learning university.

### Data collection

The data was elicited through unstructured interviews which began with a lead question regarding mobile devices and learning. Interviews lasted between 30 min and 1 h. In keeping with Glaser’s methods for grounded theory, research interviews were detailed in field notes for later analysis, rather than being recorded [33]. Data was collected until no new concepts emerged.

### Data analysis

Analysis was undertaken using the approach documented in Glaser [33] and Glaser & Strauss [32], whereby analysis occurs on four levels. On the first level, data was collected predominantly through unstructured interviews. The second level analysis occurred simultaneously with the first level and consisted of line-by-line coding to elicit the maximum amount of information from the data, without imposing assumptions onto it. This continued until no new properties emerged from the coding. As the research progressed, codes were compared in order to generate categories and their properties. At each stage of the analysis, further participant groups were identified for investigation (as mentioned previously with the differing times from graduation). This also led to questions being asked around the motivation for learning and the access to learning, to further investigate the main concern of the nurses which emerged from the data: “Maintaining competence with limited resources.” Memoing – the act of writing reflective notes – occurred throughout the research. This raised the conceptual level of the analysis until the third level of analysis occurred, where the memos were sorted, and the resultant concepts were integrated into a substantive theory. The fourth level of analysis involved comparing the developed theory with the literature. This will be expanded upon in the discussion.

### Study rigour

To ensure strict adherence to Glaser’s approach and credibility of the research process, Dr. Helen Scott of Grounded Theory Online was consulted throughout the research [35]. The investigator used a detailed memo to document her personal beliefs regarding the research area; these beliefs were then set aside so they would not colour the research findings. The product of a grounded theory is legitimised through four outcomes: “Does the theory work to explain relevant behaviour in the substantive area of the research? Does it have relevance to the people in the substantive field? Does the theory fit the substantive area? Is it readily modifiable as new data emerge?” [33]. Member checking was used to verify whether the theory explained behaviour and had relevance and fit. The Theory of Economising Learning was presented orally to two groups of registered nurses within hospitals and written copies given to other registered nurses outside of the research group. In all cases, the nurses agreed that the theory explained behaviour, and had relevance and fit. Interrogation of the Theory within the broader nursing community will ultimately determine if the theory is relevant and if the theory is modifiable over time.

## Results

Grounded theory is useful for exposing previously unknown issues and this proved to be the case in this instance. Although the interview question focused on mobile learning and how participants use mobile devices in their lives. The main issue that concerned nurses was the challenge they faced trying to maintain professional competence given the limited resources available to them in the workplace. It is through this concern and the resultant theory that it is revealed how best to use mobile devices in postgraduate continuing education.

The analysis of the data led to the development of the Theory of Economising Learning: How nurses maintain competence with limited resources. After graduation, to secure the professional learning they required, nurses needed to contribute personal resources including time, money, technology, the site of learning and prior knowledge. Nurses indicated that these resources were limited and consequently, they needed to get the best possible learning outcomes in exchange. They had to be constantly mindful that the outcomes they received justified the expenditure, hence, economising learning. The availability of these resources is different and at some level, finite for each nurse. Following is the exposition of the Theory of Economising Learning, describing the process by which nurses maintain their professional competence with finite resources.

**Fig. 1:**
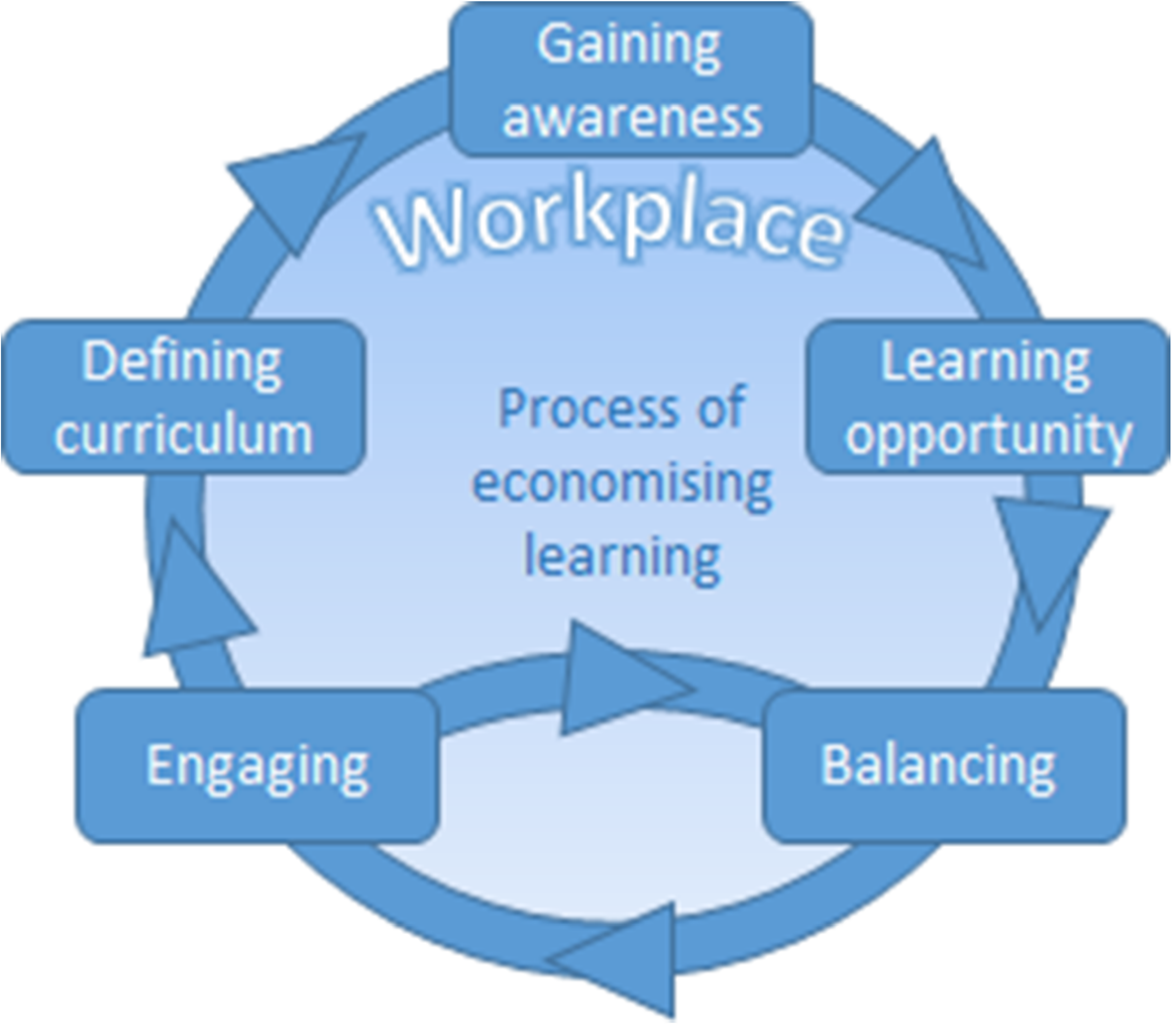
A graphical representation illustrating the stages of economising learning for nurses

The Theory of Economising Learning describes five stages of a necessarily continuous process as the nurse continually learns throughout their working life. The theory is represented in Fig. 1.

### Defining curriculum

The process starts and finishes with defining curriculum as the nurses described their awareness of the need for learning as being directly influenced by what they viewed as the knowledge needed to maintain competence in their work. Curricula in the education sector are based on what knowledge it is expected a learner will have at graduation, adequately equipping them to undertake the occupation for which they are educated. Nurses work across many different specialties and although they will require some common knowledge and skills, what the individual identifies as important to their personally identified curriculum will differ markedly. The personal curriculum is largely influenced by what is happening in a particular ward area, as one participant stated:

*I’m prompted to go to education by common themes emerging on the ward, for example renal and chest pain.*

The nurse’s personal curricula will ultimately be altered as their knowledge increases and new knowledge deficits are identified and will change depending on the area in which they work. Consequently, the next step in the process of economising learning is gaining awareness.

### Gaining awareness

Nurses described becoming aware of the need to learn in order to advance their careers, through falling short, expectations of themselves, and expectations of others. In order to advance, nurses will often become aware of their need to complete more formal learning, such as acquiring a postgraduate qualification. Examples of this would include a nurse completing postgraduate qualifications to become a Midwife or completing leadership qualifications to become a manager.

Falling short is where the nurse gains awareness of the need for further learning when their current level of knowledge compromises their competency. This can occur when they have insufficient knowledge to address a particular situation which arises in the workplace. This requires the nurse to take immediate action to maintain the safety of the patient. Two nurses discussed how they resolve this need:

*I looked up policies and procedures when I wasn’t sure how to do a procedure.*

*I go to senior staff who are really helpful telling me what I need to know.*

The research uncovered that the workplace significantly influenced whether a nurse identified the need for learning. When those in a work area have high expectations of knowledge and the acquisition of knowledge was modelled by senior staff, the participants identified that there were increased expectations around gaining knowledge. Participants also identified that the expectations of their colleagues influenced their own expectations around their knowledge. This occurred when more experienced staff placed knowledge expectations on less experienced staff or from the less experienced staff expecting that the more experienced staff would be able to answer their questions. This idea is reflected in this note from an interview:

*Learns so she has information for staff, motivated by what staff need to know and to stay current.*

The degree to which the nurses had expectations of themselves was another variable to be considered. While some nurses stated they needed sufficient knowledge to react to and understand any situation and claimed the aspiration to continuously improve their knowledge, others identified needing only the knowledge to do their job.

### Learning opportunities

Learning opportunities either flow to the nurse by being identified or offered by the organisation they work in or the nurse needs to seek out a learning opportunity. Nurses’ identified that if they needed to seek a learning opportunity it took greater personal resources and that the organisation may not support them to engage with it.

Participants described the flow of learning opportunities differently depending on their organisation and on educators within that organisation. Insufficient opportunities made it difficult for nurses to access learning as they needed to seek that learning independently, in contrast some organisations provided too many learning opportunities with many of these being mandatory. In this case the nurses used their resources to undertake the mandatory learning and became reluctant to engage with the other learning opportunities to improve their knowledge that they would ordinarily value.

An appropriate flow of learning opportunities enables nurses to not only become aware of possibilities for learning but allows them easier access to those opportunities. These opportunities can come in many different forms, including articles for informal self-directed learning, structured opportunities within the organisation, online resources or courses, or outside face-to-face workshops. The use of technology is invaluable in both facilitating learning opportunities for staff and in making staff aware of those opportunities. Technology facilitates access to repositories for learning materials, where a nurse is not only able to use a single access point to learning but is also able to target the specific learning opportunities that are relevant to them.

As the learning opportunities come into the nurse’s awareness, the nurse compares them to their personal curriculum and determines whether or not they will engage with the learning opportunities through balancing.

### Balancing

The nurses balanced their motivational factors to learn (informed by personal beliefs, the environment, extent of the knowledge deficit, expectations of self and others, and the need to move into a different position) against the personal resources (time, money, technology, the site of learning and prior knowledge) they had to contribute to that learning to determine how and if they would engage, therefore the value of the learning must outweigh the cost. The nurses also used various strategies to reduce the impact on their personal lives through moderating the amount of personal resources they needed to contribute.

When highly motivated, nurses show incredible ingenuity for finding ways to include learning in their lives. Digital technologies are often employed to save a nurse time while learning. The use of digital technologies depended on their availability, the nurse’s access to the internet and their knowledge and comfort with using technology. As a result, individuals used the technologies differently and to varying degrees to economise the resources needed to acquire knowledge. Mobile applications (“apps”) on their mobile phones or tablets were identified as being used in the workplace to enable quick access to information.

The flexibility of digital learning enabled the nurses to target the information that is relevant to them. Some nurses stated they liked to undertake online learning as they could “skim over” what they already knew and concentrate on what they did not know. As indicated in this fieldnote:

*Can learn what she wants to learn and complete in her own time, without wasting a day of her leisure time.*

Nurses often learned while multitasking. For example, nurses would listen to learning materials that had previously been downloaded to a mobile device. Nurses multitasked when waiting for appointments or waiting to pick up children from activities. Podcasts can be listened to while exercising or doing household chores. As this nurse states:

*I listen to podcasts when I’m doing the housework or mowing the lawn.*

Similar to multitasking is catching time where the nurse takes advantage of a short period of time to concentrate on learning. Catching time is often at the expense of personal time but is woven into their lives in such a way as to minimise the impact on their personal lives. The time used when catching time is usually when their families are engaged in other activities. Examples of this are when children are at after school activities or most frequently, when they are sleeping, either late at night or early in the morning. As these nurses’ state:

*After the kids go to bed I can do education.*

*I stay back after work if my son is at after school activities.*

Time can also be “caught” at work during periods when they are not so busy and at the change of shift, however the nurses reported these times as becoming less common. Both multitasking and catching time require that learning can be undertaken in small pockets of time perhaps building to a larger piece of learning.

Staying connected via social media, emails, reading journals and online groups allow nurses to undertake small amounts of learning frequently without the need to specifically search for the learning opportunity. The nurses consistently sift and sort this information and place the learning into their lives through the behaviours of multitasking, catching time and targeting.

When balancing, a nurse sometimes needed to compromise their definition of competence. Compromising occurs when the nurse’s motivation for learning is outweighed by the personal resources they need to contribute. When compromising, the nurse will not engage with a learning opportunity, therefore, balancing and engaging are dependent on each other.

### Engaging

Nurses engage with learning in one of three ways: 1) Learning on the run occurs when the nurse is engaged with work and the knowledge is needed and is immediately applied; 2) Pre-emptive learning, in advance of when it is needed, where the nurse has included the learning in their personal curriculum and may be in response to a previous knowledge deficit; and 3) credentialed learning that involves a formalised curriculum and results in a qualification. Learning on the run, pre-emptive learning, and credentialed learning are undertaken within the overall context of the workplace.

After the nurse has undertaken the process of economising learning, they will have an altered view of their learning needs and they will modify their personal curriculum accordingly. The process will then start again with the nurse’s knowledge increasing continually and the personal curriculum being altered and refined. As discussed, the process is influenced by the workplace and occurs within the context of an organisation.

### Organisation

The organisation significantly influences the nurses’ opportunities and engagement with learning. If the organisation has a culture of knowledge being valued and an expectation that nurses will use best practice, nurses indicated they would be more likely to undertake learning. Nurses also felt more inclined to use their personal resources if the organisation contributed to their learning, as one nurse explained:

*It helps when work is supportive of education. There was pressure in another hospital I worked at to do everything in your own time. Here you are encouraged more and the atmosphere is different in the hierarchy. When people apply to go to education, they usually get it and it makes you more enthusiastic to also do some in your own time.*

Access to resources within the workplace reduces the need for nurses to use their personal resources for learning. Resources include senior nurses as mentors, computers available in work areas, and time during work to undertake learning. Nurses valued senior nurses visiting them regularly during the shift and discussing patients as this education was based on the needs of their patients. One hospital had numerous computers available for staff on the ward. This meant that staff had access to them at all times, allowing them to check on things they were unsure of and to engage with learning in any downtime.

The amount of mandatory learning that a nurse needs to complete, impacts on the amount of personal resources the nurse has to allocate to learning to address knowledge deficits. Some workplaces allocated time for nurses to complete their mandatory training either by relieving them from patient care or allowing them to catch time. As this comment indicated:

*I do my internet education at work. Since there has been eLearning, I am up to date with mandatory education.*

## Discussion

It was surprising to find in this research that nurses were not concerned about the use of mobile technologies or indeed any technology to undertake learning. This has changed in recent years where previously, nurses had expressed concern about the use of technology [36]. The aim of this research was to discover how nurses were using mobile technologies in their personal and work lives, in order to leverage their existing behaviours to implement mobile learning. The aim of the research was achieved, however, nurses revealed additional information in interviews that gave insight into the decision making process to learn and how they undertake learning. It is therefore within the resultant Theory of Economising learning that the aim of the research is best situated, as the theory describes the process of how nurses decide to learn and what assists and inhibits that learning. As discussed in the background of this paper, nurses are attempting to undertake learning with limited personal resources. What has not previously been discussed in the literature, is how mobile technologies are able to save the nurse time and money by being available to them at times that suit their lives and by providing instant information where it is needed at the bedside. The findings of this research provide an opportunity for educators and workplaces to leverage the affordances of mobile technologies to equip the registered nurses with greater access to knowledge through flexible learning opportunities.

This research identified the extrinsic factors that impact on nurses balancing and engaging with education, however, it was out of scope to explicitly explore nurses’ intrinsic motivations to do so. Instead, three theories of motivation were considered to help extrapolate and explain what these motivations might be. The three theories considered here are: 1) Self-efficacy Theory [37, 38]; 2) the Theory of Planned Behaviour [39–42]; and 3) Self-Determination Theory [43, 44]. These three theories are consistent with and support the findings informing the Theory of Economising Learning in all five stages of the theory. They also explain the differences in motivation and persistence in learning between different groups of nurses. The three theories therefore add support to the validity of the Theory of Economising Learning and would be valuable for organisations to consider alongside this research.

It was identified in this research that nurses develop their own curriculum corresponding to what they see as being important knowledge in their work environment. This is impacted by extrinsic factors such as the expectations of others. It is also impacted intrinsically by their passion for the topics they are learning about and their personal search for knowledge.

Nurses can lose motivation when the organisation does not explicitly support them gaining competence, blocks their autonomy or hinders their notions of relatedness to their peers and the workplace. This is particularly evident when the learning is unreasonably mandated by the organisation, with burdensome requirements for participation that does not meet the needs of their personal curriculum. In undertaking mandated learning the individual will have fewer resources to allocate to their personal curriculum and little autonomy, reducing their ability to engage with the learning.

Nursing academic, Patricia Benner posited the Theory of Novice to Expert which was also reflected in this research [45], explaining the differing needs of nurses for expertise beyond their own in the area in which they work. The impact of experience on learning needs and how a person learns ensures that each learner will bring a unique set of experiences and expectations to every situation [46–48]. This research augments Benner’s theory by recognising that nurses’ movement between being novice and expert is fluid, depending on the situation, because of each nurse’s unique combination of knowledge and experience. Hence, in this research, knowledge is identified as one of the resources the nurse brings to their engagement with learning and has influence on their defining of curriculum.

Many of the nurses in this research were innovative in their use of mobile technologies to support their own learning. They enabled learning through using the techniques identified as multitasking, catching time, and staying connected. This is consistent with the wider literature where it is acknowledged that technologies have changed the learning landscape, allowing learning to occur outside of traditional classrooms and other physical spaces. The landscape continues to evolve at a rapid rate due to the emergence of new technologies and the corresponding changing patterns of use in relation to learning [49, 50].

## Conclusion

Though the original focus of this research was to discover how nurses used mobile technologies in their personal and professional lives, it also unearthed the processes by which nurses ensured their own competency through addressing knowledge deficits. Though their preference was to meet their continuing professional development needs during work hours, they found there were often limited resources to do so. Invested in maintaining their own competence, nurses developed a range of strategies to ensure that the learning continued, often investing their own resources including time and money. This article addresses a gap in the literature, exploring how nurses resource their own professional development, in order to maintain competence, sometimes in the absence of appropriate resourcing to do so in the workplace.

This article has described the generation of the Theory of Economising Learning to explain the continuous process by which nurses’ access and undertake learning, and where and how they do so. The process of economising learning is undertaken within the context of an organisation and involves defining a personal curriculum, gaining awareness of knowledge deficits, identifying learning opportunities, balancing opportunity with resource cost, and finally, engaging with learning. What is most significant is understanding how nurses ensure their competence using the limited resources available to them. Digital technologies are useful in this process as they are available to use in any location and at any time with nurses already owning devices and being familiar with their use. The use of online learning more generally is also valuable in that it facilitates learning without the need to travel to a campus or other physical location, while still enabling the nurse to choose something that is relevant to their own needs.

This substantiative grounded theory, the Theory of Economising Learning, can only be applied to the area where the research was conducted. This research is therefore only applicable to registered nurses within Australia. It is possible, and perhaps even probable, that the Theory of Economising Learning could explain the learning behaviours of nurses in other countries or be applicable to other professions, however, theoretical sampling of those wider groups would need to occur to confirm emergent fit. Even so, there is much to be gained by incorporating the learnings of this research when planning for continuing professional development for nurses in the context in which the research took place.

Maintaining the competence of nurses in the workplace through continuing professional development is more likely to be successful if certain strategies are adopted. This research posits that workplaces need to create reasonable expectations within nurses to address knowledge deficits and provide the resources, including time, to allow them to do so without significant personal cost. It is also necessary for workplaces to moderate the flow of learning opportunities so as not to overwhelm and demotivate the nurses. Currently, nurses use several strategies to optimise their learning using mobile technologies. These could be leveraged in the workplace to help nurses maintain professional competence.

With health care settings encountering a range of emerging challenges such as with the COVID-19 pandemic, the need for the health workforce to be agile in terms of knowledge, has never been greater. Careful planning around the delivery of that learning, including the resourcing of it, means of delivery and creating the expectation of engagement is necessary to ensure its success.

### Limitations

This study has been conducted with postgraduate nurses within Australia, it is therefore not known if the theory could be generalised across the rest of the world and to other professions. The broader literature in nursing, however, is consistent with the findings of this study and it is therefore likely that the findings will be applicable outside the group studied. Further research would need to be conducted to determine if the findings could be generalised outside of the nursing profession, as it not known if other professions face the same issues in regard to professional development.

Care was taken within this research to ensure that a wide variety of nurses were interviewed, and that nurses of all levels of comfort with technology were recruited. It is possible however, that those that self-selected to participate in the research were those nurses that display driven behaviours toward learning and also those that are more interested in using mobile devices.

## Data Availability

Data collected for this research is not publicly available as it could compromise participants or the organisation where they are employed.

## Abbreviations

Apps: Mobile applications
